# Deciding the Bisimilarity of Context-Free Session Types

**DOI:** 10.1007/978-3-030-45237-7_3

**Published:** 2020-03-13

**Authors:** Bernardo Almeida, Andreia Mordido, Vasco T. Vasconcelos

**Affiliations:** 8 Johannes Kepler University, Linz, Austria; 9 University of Birmingham, Birmingham, UK; LASIGE, Faculdade de Ciências, Universidade de Lisboa, Lisbon, Portugal

**Keywords:** Types, Type equivalence, Bisimulation, Algorithm

## Abstract

We present an algorithm to decide the equivalence of context-free session types, practical to the point of being incorporated in a compiler. We prove its soundness and completeness. We further evaluate its behaviour in practice. In the process, we introduce an algorithm to decide the bisimilarity of simple grammars.

## 1 Introduction

Session types enhance the expressivity of traditional types for programming languages by allowing the description of structured communication on heterogeneously typed channels [14,15,24]. Traditional session types are *regular* in the sense that the sequences of communication actions admitted by a type are in the union of a regular language (for finite executions) and an $\omega$-regular language (for infinite executions). Introduced by Thiemann and Vasconcelos, context-free session types liberate traditional session types from the shackles of tail recursion, allowing, for example, the safe serialization of arbitrary recursive datatypes [26].

Session types are often used to discipline interactions in concurrent programs. When associated to (bidirectional, heterogeneous) channels, session types describe the permitted patterns of interaction. For example, a type of the form

```
rec x. +{Leaf: Skip, Node: !Int;x;x}
```

may describe one end of a communication channel. A process holding such a channel end must first *select* between choices Leaf and Node. If Leaf is chosen, then type Skip forwards the interaction to the continuation, if any. If no continuation is present, then interaction is over. Otherwise, the process must send an integer (!Int) followed by two trees, as witnessed by the recursive calls occurring after the choice of Node. A concurrent process holding the other end of the channel interacts via a *dual* type:

```
rec y. &{Leaf: Skip, Node: ?Int;y;y}
```

In this case the process must be ready to offer both choices, Leaf and Node. For the latter option, the process must further receive an integer (?Int), followed by two trees.

Regular languages cannot capture such behaviour. The best one can do with regular session types (and without resorting to channel passing) is to use a regular type that allows transmitting trees, as well as many other non tree-like structures. The correct behaviour of processes interacting on such a channel would need to be checked at runtime [2,26].

If the algorithmic aspects of type equivalence for regular session types are well known (Gay and Hole propose an algorithm to decide subtyting [9], from which type equivalence can be derived), the same does not apply to context-free session types. Thiemann and Vasconcelos [26] show that the equivalence of context-free session types is decidable, by reducing the problem to the verification of bisimulation for Basic Process Algebra (BPA) which, in turn, was proved decidable by Christensen, Hüttel, and Stirling [6]. Even if the equivalence problem for context-free session types is known to be decidable, no algorithm has been proposed. Padovani [20] introduces a language with context-free session types that avoids the problem of checking the equivalence of types by requiring annotations in the source code. Annotations result in the structural alignment between code and types. This alignment—enforced by an explicit resumption process operator that breaks sequential composition in types—sidesteps the problem central to this paper: that of checking type equivalence. Furthermore, there are some basic equivalences on types that the compiler is not able to identify [20].

After the breakthrough by Christensen, Hüttel, and Stirling—a result that provides no immediate practical algorithm—the problem of deciding the equivalence of BPA terms has been addressed by several researchers [4,6,8,18]. Most of these works provide no practical algorithm that can be readily used, except the one by Czerwinski and Lasota where a polynomial time algorithm is presented that decides the bisimilarity of normed context-free processes in $\mathcal{O}(n^5)$ [8]. However, context-free session types are not necessarily normed, which precludes resorting to this algorithm, or using the original result by Baeten, Bergstra, and Klop [3], as well as improvements by Hirshfeld, Jerrum, and Moller [12,13]. Moreover, the complexity estimates for deciding bisimilarity in BPA process are not promising. Kiefer provided an EXPTIME lower bound for BPA bisimilarity by proving this problem is EXPTIME-hard [19], whereas Jančar has provided a double exponential upper bound for this problem and proved that its complexity is $\mathcal{O}(2^{2^{\mathsf{pol}(n)}})$ [17].

The decidability of deterministic pushdown automata (DPDA) has also been subject of much study [16,22,23]. Several techniques have been proposed to solve the problem, but no immediate practical algorithm was available until Henry and Sénizergues provide an algorithm for this problem [10]. Its poor performance however precludes its incorporation in a compiler. Furthermore, the algorithm Henry and Sénizergues propose handles the problem of language equivalence rather than the problem of deciding bisimilarity of DPDAs.

Our algorithm to decide the equivalence of context-free session types also allows deciding the bisimilarity of simple grammars (i.e., deterministic grammars in Greibach Normal Formal). It proceeds in three stages. The *first stage* builds a context-free grammar in Greibach Normal Formal (GNF)—in fact a simple grammar—from two context-free session types in a way that bisimulation is preserved. A basic result from Baeten, Bergstra, and Klop states that any guarded BPA system can be transformed into Greibach Normal Formal (GNF) while preserving bisimulation equivalence, but unfortunately no procedure is presented [3]. The *second stage* prunes the grammar by removing unreachable symbols in unnormed sequences of non-terminal symbols. This stage builds on the result of Christensen, Hüttel, and Stirling [6]. The *third stage* constructs an expansion tree, by alternating between expansion and simplification steps. This last stage uses expansion operations proposed by Jančar, Moller, and Hirshfeld [11,18], and simplification rules proposed by Caucal, Christensen, Hüttel, Stirling, Jančar, and Moller [5,6,18]. The finite representation of bisimulations of BPA transition graphs [5,6] is paramount for our results of soundness and completeness.

The branching nature of the expansion tree confers an (at least) exponential complexity to the algorithm. However, our experiments with a concrete implementation—both as a stand-alone tool and incorporated in a compiler [2]—are promising. We propose heuristics that decrease the execution time in 89% and reduce the number of timeouts by 95% (see Section 5).

We present an algorithm to decide the equivalence of context-free session types, practical to the point of being readily included in any compiler, an exercise that we conducted in parallel [2]. The main contributions of this work are:

– The proposal and implementation of an algorithm to decide type equivalence of context-free session types;
– A proof of its soundness and completeness against the declarative definition;
– The proposal and implementation of an algorithm to decide the bisimilarity of simple grammars; and
– The empirical study of the runtime behaviour of the implementation.

The rest of the paper is organized as follows: an introduction to context-free session types can be found in Section 2, the algorithm in Section 3, the main results in Section 4, evaluation in Section 5, and conclusions in Section 6.

## 2 Context-free session types

This section briefly introduces context-free session types, based on the work of Thiemann and Vasconcelos [26]. The types we consider build upon a denumerable set of *variables* and a set of *choice labels*. Metavariables $X, Y, Z$ range over variables and $\ell$ over labels. We assume given a set of base types denoted by $B$. The syntax of types is given by the grammar below.

$$S, T ::= \mathsf{skip} \mid \sharp B \mid \star\{\ell_i \colon T_i\}_{i\in I} \mid S;T \mid \mu X.T \mid X$$
$$\sharp ::= ! \mid ? \qquad\qquad \star ::= \oplus \mid \&$$

In type $\mu X.T$, variable $X$ is bound in the subterm $T$. The sets of bound and free variables in a given type are defined accordingly. Notation $[T/X]S$ denotes the resulting of substituting T for the (free) occurrences of $X$ in $S$.

Judgement $S\checkmark$ characterizes *terminated* types: context-free session types that exhibit no further action [1].

Terminated predicate: $\boxed{T\checkmark}$

$$\mathsf{skip}\checkmark \qquad X\checkmark \qquad \frac{S\checkmark \quad T\checkmark}{S;T\checkmark} \qquad \frac{T\checkmark}{\mu X.T\checkmark}$$

Notice that all types of the form $\mu X.\mu X_1 \dots \mu X_n.X$, for $n \geq 0$, are terminated.

We are not interested in all types generated by the above grammar. If $\Delta$ is a list of pairwise distinct variables, then judgement $\Delta \vdash T$ characterises the types of interest: the *well-formed* types.

Type formation system: $\boxed{\Delta \vdash T}$

$$\frac{}{\Delta \vdash \mathsf{skip}} \qquad \frac{}{\Delta \vdash \sharp B} \qquad \frac{X \in \Delta}{\Delta \vdash X} \qquad \frac{\Delta \vdash S \quad \Delta \vdash T}{\Delta \vdash S;T} \qquad \frac{\Delta \vdash T_i \quad (\forall i \in I)}{\Delta \vdash \star\{\ell_i \colon T_i\}_{i\in I}} \qquad \frac{\neg T\checkmark \quad \Delta, X \vdash T}{\Delta \vdash \mu X.T}$$

Terminated processes have a simple characterisation—types comprising skip, $\mu$ and semicolon—which justifies the inclusion of $\neg T\checkmark$ in the rules for type formation (Thiemann and Vasconcelos [26] introduce a contractive judgement for the effect). Type formation serves two main purposes: ensuring that all variables introduced by $\mu$-types are pairwise distinct and that types underneath a $\mu$ are not terminated. This can be clearly seen by formation rule for $\mu$-types, where notation $\Delta, X$ is understood as requiring $X \notin \Delta$. In the sequel we assume that all types are such that $\vdash T$ and denote by $\mathcal{T}$ the set such types.

The set of *actions* is generated by the following grammar.

$$a \; ::= \; \sharp B \;\mid\; \star\ell$$

The *labelled transition system* (LTS) for context-free session types is given by $\mathcal{T}$ as the set of *states*, the set of *actions*, and the *transition relation* $S \xrightarrow{a}_{\mathcal{T}} T$ defined by the rules below.

Labelled transition system: $\boxed{S \xrightarrow{a}_{\mathcal{T}} T}$

$$\sharp B \xrightarrow{\sharp B}_{\mathcal{T}} \mathsf{skip} \qquad \star\{\ell_i \colon T_i\}_{i\in I} \xrightarrow{\star\ell_j}_{\mathcal{T}} T_j \quad (j \in I)$$

$$\frac{S \xrightarrow{a}_{\mathcal{T}} S'}{S;T \xrightarrow{a}_{\mathcal{T}} S';T} \qquad \frac{S\checkmark \quad T \xrightarrow{a}_{\mathcal{T}} T'}{S;T \xrightarrow{a}_{\mathcal{T}} T'} \qquad \frac{[\mu X.S/X]S \xrightarrow{a}_{\mathcal{T}} T}{\mu X.S \xrightarrow{a}_{\mathcal{T}} T}$$

*Type bisimulation* is defined in the usual way from the labelled transition system [21]. We say that a type relation $\mathcal{R}$ is a *bisimulation* if, whenever $S\mathcal{R}T$, for all $a$ we have:

- for each $S'$ with $S \xrightarrow{a}_{\mathcal{T}} S'$, there is $T'$ such that $T \xrightarrow{a}_{\mathcal{T}} T'$ and $S'\mathcal{R}T'$, and
- for each $T'$ with $T \xrightarrow{a}_{\mathcal{T}} T'$, there is $S'$ such that $S \xrightarrow{a}_{\mathcal{T}} S'$ and $S'\mathcal{R}T'$.

We say that two types are bisimilar, written $S \sim_{\mathcal{T}} T$, if there is a bisimulation $\mathcal{R}$ with $S\mathcal{R}T$.

## 3 An algorithm to decide type bisimilarity

This section presents an algorithm to decide whether two types are in a type bisimulation. In the process we also provide an algorithm to decide the bisimilarity of simple context-free languages. The algorithm comprises three stages:

1. Translate the two types to a simple grammar,
2. Prune unreachable symbols, and
3. Explore an expansion tree, alternating between simplification and expansion operations, until finding an empty node—case in which it decides positively—or failing to expand all nodes—case in which it decides negatively.

*Translating types to grammars.* Type variables $X$ are the *non-terminal symbols* and LTS labels $a$ are the *terminal symbols*. Sequences of type variables $\vec{X}$ are called *words*; $\varepsilon$ denotes the empty word. A context-free grammar in Greibach Normal Form is a pair $(\vec{X}, \mathcal{P})$ where $\vec{X}$ is the *start word* and $\mathcal{P}$ a *set of productions* of the form $Y \to a\vec{Z}$ (context-free session types do not require productions of the form $Y \to \varepsilon$). Due to the deterministic nature of context-free session types, the grammars we are interested in are *simple*: for each non-terminal symbol $Y$ and terminal symbol $a$, there is at most one production of the form $Y \to a\vec{Z}$.

Grammars in Greibach normal form naturally induce a labelled transition system by taking words $\vec{X}$ for states, terminal symbols $a$ for actions, and $\xrightarrow{a}_{\mathcal{P}}$, defined as $X\vec{Y} \xrightarrow{a}_{\mathcal{P}} \vec{Z}\vec{Y}$ when $X \to a\vec{Z} \in \mathcal{P}$, for the transition relation. The associated bisimilarity is denoted by $\sim_{\mathcal{P}}$.

The *unravelling* function on well-formed context-free session types, taken from Thiemann and Vasconcelos [26], is defined as follows.

$$\begin{aligned}
\mathsf{unr}(\mu X.T) &= \mathsf{unr}([\mu X.T/X]T) \\
\mathsf{unr}(S;T) &= \begin{cases} \mathsf{unr}(T) & \mathsf{unr}(S) = \mathsf{skip} \\ (\mathsf{unr}(S);T) & \mathsf{unr}(S) \neq \mathsf{skip} \end{cases} \\
\mathsf{unr}(T) &= T \qquad \text{in all other cases}
\end{aligned}$$

The function terminates under the assumption that types are well formed.

Another function, word, builds a word from a type. In the process it updates a global set $\mathcal{P}$ of grammar productions. Word concatenation is denoted by $\vec{X} \cdot \vec{Y}$.

$$\begin{aligned}
\mathsf{word}(\mathsf{skip}) &= \varepsilon \\
\mathsf{word}(S;T) &= \mathsf{word}(S) \cdot \mathsf{word}(T) \\
\mathsf{word}(\sharp B) &= Y, \text{ setting } \mathcal{P} := \mathcal{P} \cup \{Y \to \sharp B\} && (Y \text{ fresh}) \\
\mathsf{word}(\star\{\ell_i \colon T_i\}_{i\in I}) &= Y, \text{ setting } \mathcal{P} := \mathcal{P} \cup \{Y \to \star\ell_i \cdot \mathsf{word}(T_i) \mid i \in I\} && (Y \text{ fresh}) \\
\mathsf{word}(X) &= X \\
\mathsf{word}(\mu X.T) &= X
\end{aligned}$$

The following lemma relates terminated types to the result of a call to word.

**Lemma 1.** *Let* $\vdash T$. *Then,* $T\checkmark$ *if and only if* $\mathsf{word}(T) = \varepsilon$.

*Proof.* The direct implication follows by rule induction on predicate $\checkmark$:

- Case $\mathsf{skip}\checkmark$: $\mathsf{word}(\mathsf{skip}) = \varepsilon$.
- Case $X\checkmark$: if $T$ is $X$, then $\nvdash T$.
- Case $S;T\checkmark$: by induction hypothesis on the rule premises $S\checkmark$ and $T\checkmark$, $\mathsf{word}(S) = \varepsilon$ and $\mathsf{word}(T) = \varepsilon$. Hence, $\mathsf{word}(S;T) = \varepsilon$.
- Case $\mu X.S$: the hypothesis $T\checkmark$ and the rule premises of hypothesis $\vdash T$ are contradictory.

Conversely, if $\mathsf{word}(T) = \varepsilon$, using the rules of the definition of $\mathsf{word}$ that produce the empty word:

- if $T$ is $\mathsf{skip}$, then we have $T\checkmark$.
- if $T$ is $U;V$, $\mathsf{word}(U) = \varepsilon$, and $\mathsf{word}(V) = \varepsilon$, then, by induction, we have $U\checkmark$ and $V\checkmark$. Hence, $T\checkmark$.
- No other case in function $\mathsf{word}$ produces an empty word. □

To define the translation of context-free session types to simple grammars, assume that $\{\mu X_1.T_1, \ldots, \mu X_n.T_n\}$ is the set of all $\mu$-subterms in a given type $T$. Further assume that $i < j$ whenever $X_j \in \mathsf{free}(\mu X_i.T_i)$. That is, the $\mu$-subterms are topologically sorted with respect to their lexical nesting, innermost subterms first. Now we identify unrolled versions of the $\mu$-subterms.

$$
\begin{aligned}
T_1' &= [\mu X_n.T_n/X_n] \cdots [\mu X_2.T_2/X_2][\mu X_1.T_1/X_1]T_1 \\
T_2' &= [\mu X_n.T_n/X_n] \cdots [\mu X_2.T_1/X_2]T_2 \\
&\ \vdots \\
T_n' &= [\mu X_n.T_n/X_n]T_n
\end{aligned}
$$

Clearly each type $T_i'$ is closed (has no free variables). Notice that if $T$ is a $\mu$-type, then $\mu X_n.T_n$ is T itself.

Finally, given an initial set of productions $\mathcal{P}_0$, function $\mathsf{grm}$ translates a type $T$ into a grammar composed of a start word and set of productions:

$$\mathsf{grm}(T, \mathcal{P}_0) = (\mathsf{word}(T), \mathcal{P}_n)$$

where each $\mathcal{P}_i$ is computed from $\mathcal{P}_{i-1}$ by the following recurrence,

$$\mathcal{P}_i' \cup \{X_i \to a_j \vec{Y}_j \vec{Z} \mid (Z \to a_j \vec{Y}_j) \in \mathcal{P}_i'\} \text{ where } (Z\vec{Z}, \mathcal{P}_i') = \mathsf{grm}(\mathsf{unr}(T_i'), \mathcal{P}_{i-1})$$

Notice that $\mathsf{word}(\mathsf{unr}(T_i'))$ is a non-empty word because of Lemma 1 and the fact that each $T_i'$ is non-terminated by hypothesis. The function $\mathsf{grm}$ terminates on all inputs (because recursion is always on subterms) and adds a finite number of productions to the original set. Furthermore, because choices in session types do not contain duplicated labels, the function returns a simple grammar.

To run $\mathsf{grm}$ on two well-formed types proceed as follows: rename the second type so that bound variables do not overlap with those of the first; start with an empty set of productions; run the algorithm consecutively on the two types to obtain two initial words and a single set of productions.

*Example 1.* Consider the following pair of context-free session types.

$$S \triangleq (\mu X_1.\&\{n : X_1; X_1; ?\mathsf{int}, \ell : ?\mathsf{int}\}); (\mu X_2.!\mathsf{int}; X_2; X_2)$$
$$T \triangleq (\mu Y_1.\&\{n : Y_1; Y_1, \ell : \mathsf{skip}\}; ?\mathsf{int}); (\mu Y_2.!\mathsf{int}; Y_2)$$

Starting from the empty set of productions, running grm consecutively on $S$ and on $T$ produces the following set of productions

$$\begin{array}{llll} X_1 \to \&n\, X_1X_1X_3 & X_3 \to ?\mathsf{int} & Y_1 \to \&n\, Y_1Y_1Y_3 & Y_2 \to !\mathsf{int}\, Y_2 \\ X_1 \to \&\ell\, X_4 & X_4 \to ?\mathsf{int} & Y_1 \to \&\ell\, Y_3 & Y_3 \to ?\mathsf{int} \\ X_2 \to !\mathsf{int}\, X_2X_2 & & & \end{array}$$

and two start words $X_1X_2$ and $Y_1Y_2$.

*Pruning unnormed productions.* For $\vec{a}$ a non-empty sequence of non-terminal symbols $a_1, \ldots, a_n$, write $\vec{Y} \xrightarrow{\vec{a}}_{\mathcal{P}} \vec{Z}$ when $\vec{Y} \xrightarrow{a_1}_{\mathcal{P}} \cdots \xrightarrow{a_n}_{\mathcal{P}} \vec{Z}$. We say that $\vec{Y}$ is *normed* when $\vec{Y} \xrightarrow{\vec{a}}_{\mathcal{P}} \varepsilon$ for some $\vec{a}$, and that $\vec{Y}$ is *unnormed* otherwise. When $\vec{Y}$ is normed, the *minimal path* of $\vec{Y}$ is the shortest $\vec{a}$ such that $\vec{Y} \xrightarrow{\vec{a}}_{\mathcal{P}} \varepsilon$. In this case, the *norm* of $\vec{Y}$, denoted by $|\vec{Y}|$, is the length of $\vec{a}$. As observed by Christensen, Hüttel, and Stirling [6], any unnormed word $\vec{Y}$ is bisimilar to its concatenation with any other word, that is, if $\vec{Y}$ is unnormed, then $\vec{Y} \sim_{\mathcal{P}} \vec{Y}\vec{X}$. We use this fact to prune unreachable symbols in unnormed words. And we do this in all productions.

*Example 2.* Recall Example 1 and notice that $X_2$ and $Y_2$ are both unnormed. Then, the last occurrence of $X_2$ in production $X_2 \to !\mathsf{int}\, X_2X_2$ is unreachable, hence we simplify the production to obtain $X_2 \to !\mathsf{int}\, X_2$.

*Building an expansion tree.* We base the third stage of the algorithm on the notion of *expansion tree* as proposed by Jančar and Moller [18], adapting an idea by Hirshfeld [11]. The *nodes* in trees are labelled by sets of pairs of words. We say that a node $N'$ is an *expansion* of $N$ if $N'$ is a minimal set such that: for every pair $(\vec{X}, \vec{Y}) \in N$,

– if $\vec{X} \to a\vec{X}'$ then $\vec{Y} \to a\vec{Y}'$ with $(\vec{X}', \vec{Y}') \in N'$, and
– if $\vec{Y} \to a\vec{Y}'$ then $\vec{X} \to a\vec{X}'$ with $(\vec{X}', \vec{Y}') \in N'$.

An *expansion tree* is built from a root node: the singleton set containing the pair of start words obtained by translating the two types into a grammar. A children node is obtained from its parent node by expansion. However, as Jančar and Moller observed, expansions alone often lead to infinite trees. We then alternate between expansion and simplification operations, until either finding an empty node—case in which we decide equivalence positively—or failing to expand all nodes—case in which we decide equivalence negatively. We say that a branch is *successful* if it is infinite or finishes in an empty node, otherwise it is said to be *unsuccessful*.

In the *expansion step*, each node $N$ derives a single child node, obtained as an expansion of $N$. As we are dealing with simple grammars, no branching is expected in the expansion tree at this step.

The *simplification step* consists on the application of the following rules:

**Reflexive rule:** Omit from a node any pair of the form $(\vec{X}, \vec{X})$;
**Congruence rule:** Omit from a node $N$ any pair that belongs to the least congruence containing the ancestors of $N$;
**BPA1 rule:** If $(X_0\vec{X}, Y_0\vec{Y})$ is in $N$ and $(X_0\vec{X'}, Y_0\vec{Y'})$ belongs to the ancestors of $N$, then create a sibling node for $N$ replacing $(X_0\vec{X}, Y_0\vec{Y})$ by $(\vec{X}, \vec{X'})$ and $(\vec{Y}, \vec{Y'})$;
**BPA2 rule:** If $(X_0\vec{X}, Y_0\vec{Y})$ is in $N$ and $X_0$ and $Y_0$ are normed, then:
- **Case $|X_0| \leq |Y_0|$:** Let $\vec{a}$ be a minimal path for $X_0$ and $\vec{Z}$ the word such that $Y_0 \xrightarrow{\vec{a}}_{\mathcal{P}} \vec{Z}$. Add a sibling node for $N$ including the pairs $(X_0\vec{Z}, Y_0)$ and $(\vec{X}, \vec{Z}\vec{Y})$ in place of $(X_0\vec{X}, Y_0\vec{Y})$;
- **Otherwise:** Let $\vec{a}$ be a minimal path for $Y_0$ and $\vec{Z}$ the word such that $X_0 \xrightarrow{\vec{a}}_{\mathcal{P}} \vec{Z}$. Add a sibling node for $N$ including the pairs $(X_0, Y_0\vec{Z})$ and $(\vec{Z}\vec{X}, \vec{Y})$ in place of $(X_0\vec{X}, Y_0\vec{Y})$.

Contrarily to expansion and to the reflexive and congruence simplifications, BPA rules promote branching in the expansion tree. We iteratively apply the simplification rules to ensure the algorithm computes the simplest possible children nodes derived from $N$. We can easily show that the simplification function that results from applying the reflexive, congruence, and BPA rules, has a fixed point in the complete partial ordered set of pairs node-ancestors, where the set of ancestors is fixed. The proof builds a partial order on the sets of pairs node-ancestors and uses Tarski's fixed point theorem [25]. The number of children nodes generated by the application of these rules is finite [6,18]. Notice that the sibling nodes do not exclude the (often) infinite branch resulting from successive expansions.

*Checking the bisimilarity of simple grammars.* Given a set of productions and two start words $\vec{X}$ and $\vec{Y}$ (all pruned), function bisimG alternates between simplification and expansion stages, starting with expansion. To avoid getting stuck in an infinite branch of the expansion tree, we use a breadth-first search on the expansion tree: node-ancestor pairs to be processed are stored in a queue. The initial pair inserted in the queue contains the initial node $\{(\vec{X}, \vec{Y})\}$ and an empty set of ancestors.

$$\mathsf{bisimG}(\vec{X}, \vec{Y}, \mathcal{P}) = \mathsf{expand}(\mathsf{singletonQueue}((\{(\vec{X}, \vec{Y})\}, \emptyset), \mathcal{P})$$

Predicate expand terminates as soon as all nodes fail to expand (signalled by an empty queue), case in which the algorithm returns **False**, or an empty node is reached, case in which the algorithm returns **True**. Otherwise, it extracts node

$n$ at the front of the queue, simplifies its child node, and recurs.

$$
\begin{array}{l}
\mathsf{expand}(q, \mathcal{P}) = \\
\quad \textbf{if } \mathsf{empty}(q) \textbf{ then False} \\
\quad \textbf{else } (n, a) = \mathsf{front}(q) \\
\qquad \textbf{if } \mathsf{empty}(n) \textbf{ then True} \\
\qquad \textbf{else if } \mathsf{hasChild}(n, \mathcal{P}) \\
\qquad\quad \textbf{then } \mathsf{expand}(\mathsf{simplify}(\{(\mathsf{child}(n, \mathcal{P}), a \cup n)\}, \mathsf{dequeue}(q), \mathcal{P})) \\
\qquad\quad \textbf{else } \mathsf{expand}(\mathsf{dequeue}(q), \mathcal{P})
\end{array}
$$

The simplification stage distinguishes the case where all type variables are normed, in which case BPA1 is not required to decide equivalence [5,6], from the case where some type variables might be unnormed.

$$
\begin{array}{l}
\mathsf{rules} = \textbf{if } \mathsf{allProductionsNormed}(\mathcal{P}) \textbf{ then } [\mathsf{reflex}, \mathsf{congruence}, \mathsf{bpa2}] \\
\qquad\qquad \textbf{else } [\mathsf{reflex}, \mathsf{congruence}, \mathsf{bpa1}, \mathsf{bpa2}]
\end{array}
$$

Function simplify applies the various rules iteratively, until reaching a fixed point. The application of the rules (via function apply) produces a set of nodes that are then enqueued. The simplification stage does not introduce new levels in the tree, hence the set of ancestors $na$ is passed to function apply as is.

$$\mathsf{simplify}(na, q, \mathcal{P}) = \textbf{fold}(\mathsf{enqueue}, q, \mathsf{apply}(na, \mathsf{rules}, \mathcal{P}))$$

*Example 3.* The expansion tree for our running example is in Figure 1. Once a successful branch is reached (marked with ✓), $\mathsf{bisimG}(\vec{X}, \vec{Y}, \mathcal{P})$ returns **True**.

*Checking the bisimilarity of context-free session types.* Function bisimT decides the equivalence of two well-formed and renamed types, $S$ and $T$. It starts by computing the start words for $S$ and $T$ by first translating $S$ to a grammar and enriching this with the productions for type $T$. After pruning the productions in the grammar (function prune), the equivalence of $S$ and $T$ is decided using function bisimG.

$$
\begin{array}{rl}
\mathsf{bisimT}(T, U) = \mathsf{bisimG}(\vec{X}, \vec{Y}, \mathsf{prune}(\mathcal{P})) & \\
\textbf{where } (\vec{X}, \mathcal{P}') = & \mathsf{grm}(S, \emptyset) \\
(\vec{Y}, \mathcal{P}) = & \mathsf{grm}(T, \mathcal{P}')
\end{array}
$$

## 4 Correctness of the algorithm

In this section we prove that function bisimT is sound and complete with respect to the meta-theory of context-free session types. We start by showing a full abstraction result between context-free session types and grammars in Greibach Normal Form. Then, based on results from Caucal [5], Christensen, Hüttel, and Stirling [6], Jančar and Moller [18], we conclude that the algorithm we propose is sound and complete.

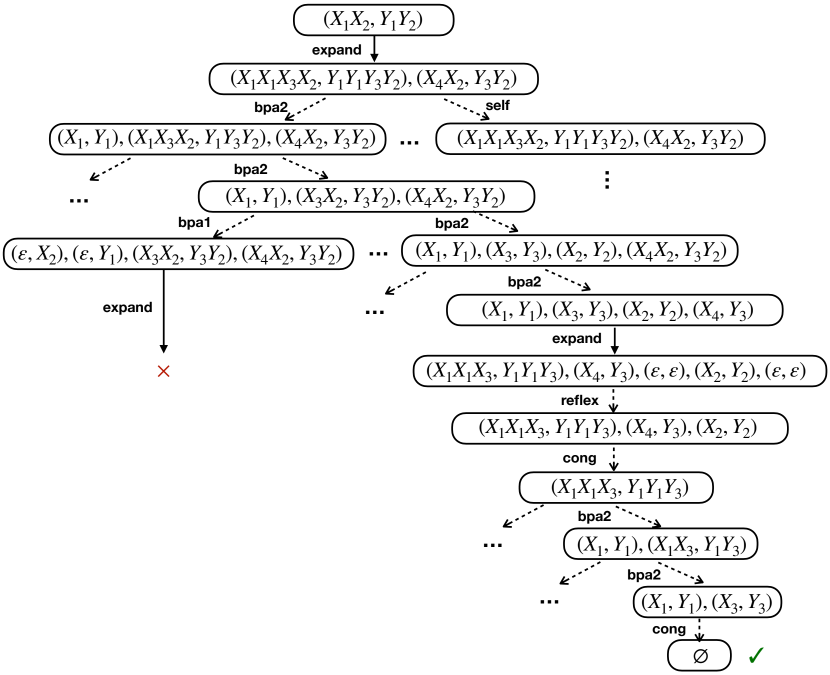

**Fig. 1:** An example of an expansion tree

*Type translation is fully abstract.* Sections 2 and 3 introduce bisimulation relations on the set $\mathcal{T}$ of types $\sim_\mathcal{T}$ and on a given set $\mathcal{P}$ of productions $\sim_\mathcal{P}$. Our ultimate goal is to prove that we can faithfully analyze the bisimilarity of types by analyzing the bisimilarity of the corresponding grammars. For this purpose, we prove that the translation proposed in Section 3 is a *fully abstract encoding*, i.e., preserves the bisimilarity relation.

We start showing that the transformation of types to grammars preserves the labelled transitions. The following result states that grammars produced by $\mathsf{grm}$ mimic the transitions of the corresponding types and vice-versa.

**Lemma 2.** *Let* $(\vec{X}, \mathcal{P}') = \mathsf{grm}(S, \emptyset)$ *and* $(\vec{Y}, \mathcal{P}) = \mathsf{grm}(T, \mathcal{P}')$*. Then,* $S \xrightarrow{a}_\mathcal{T} T$ *if and only if* $\vec{X} \xrightarrow{a}_\mathcal{P} \vec{Y}$*.*

*Proof.* For the direct implication we proceed by rule induction on the hypothesis, using the definition of $\mathsf{word}$.

- Case $\sharp B$: if $\sharp B \xrightarrow{\sharp B}_\mathcal{T} \mathsf{skip}$, then $\mathsf{word}(\sharp B) \xrightarrow{\sharp B}_\mathcal{P} \varepsilon$.
- Case $\star\ell_i$: if $\star\{\ell_i\colon S_i\}_{i\in I} \xrightarrow{\star\ell_i}_\mathcal{T} S_i$, then $\mathsf{word}(S) \xrightarrow{\star\ell_i}_\mathcal{P} \mathsf{word}(S_i)$.
- Case $S_1;S_2$ with $S_1 \xrightarrow{a}_\mathcal{T} S_1'$: if $S_1;S_2 \xrightarrow{a}_\mathcal{T} S_1';S_2$ and $S_1 \xrightarrow{a}_\mathcal{T} S_1'$, by induction hypothesis, we have $\mathsf{word}(S_1) \xrightarrow{a}_\mathcal{P} \mathsf{word}(S_1')$. Furthermore, $\mathsf{word}(S_1;S_2) = \mathsf{word}(S_1)\cdot\mathsf{word}(S_2)$. Hence, $\mathsf{word}(S_1;S_2) \xrightarrow{a}_\mathcal{P} \mathsf{word}(S_1';S_2)$.

– Case $S_1; S_2$ with $S_1\checkmark$ and $S_2 \xrightarrow{a}_{\mathcal{T}} S_2'$: in the case $S_1; S_2 \xrightarrow{a}_{\mathcal{T}} S_2'$, where $S_1\checkmark$ and $S_2 \xrightarrow{a}_{\mathcal{T}} S_2'$, by Lemma 1 and since $S_1\checkmark$, we have $\mathsf{word}(S_1; S_2) = \mathsf{word}(S_2)$. Thus, by induction hypothesis we have $\mathsf{word}(S_1; S_2) \xrightarrow{a}_{\mathcal{P}} \mathsf{word}(S_2')$.
– Case $\mu X.T$: if $\mu X.T \xrightarrow{a}_{\mathcal{T}} S'$, then $[\mu X.T/X]T \xrightarrow{a}_{\mathcal{T}} S'$. Also $\mathsf{unr}(S) \xrightarrow{a}_{\mathcal{T}} S'$ and, by induction hypothesis, $\mathsf{word}(\mathsf{unr}(S)) \xrightarrow{a}_{\mathcal{P}} \mathsf{word}(S')$. Hence, by definition of $\mathsf{word}$, $\mathsf{word}(S) = X \xrightarrow{a}_{\mathcal{P}} \mathsf{word}(S')$.

For the reverse implication, we prove that any transition in the grammar leads to a transition in the corresponding types.

– if $\mathsf{word}(S) \xrightarrow{\sharp B}_{\mathcal{P}} \vec{X}$, then $\mathsf{word}(S) = Y \cdot \vec{X}$, where $Y \xrightarrow{\sharp B}_{\mathcal{P}} \varepsilon$, and so $\mathsf{unr}(S) = \sharp B; T$ and thus $S \xrightarrow{\sharp B}_{\mathcal{T}} T$.
– if $\mathsf{word}(S) \xrightarrow{\star \ell_i}_{\mathcal{P}} \vec{X}$, then $\mathsf{word}(\mathsf{unr}(S)) = Y$, where $Y \xrightarrow{\star \ell_i}_{\mathcal{P}} \vec{X}$. Hence, $\mathsf{unr}(S)$ is of the form $\star\{\ell_j' : U_j\}_{j\in J}; T$ with $\ell_i = \ell_j'$ and $\vec{X} = \mathsf{word}(U_j; T)$, for some $j \in J$. Using the LTS we conclude that $S \xrightarrow{\star \ell_i}_{\mathcal{T}} U_j; T$. □

**Lemma 3.** *If* $\mathsf{word}\ S \xrightarrow{a}_{\mathcal{P}} \vec{X}$, *then exists* $T$ *s.t.* $S \xrightarrow{a}_{\mathcal{T}} T$ *and* $\vec{X} = \mathsf{word}\ T$.

*Proof.* By induction on the definition of $\mathsf{word}$. □

The main result of this subsection follows from Lemmas 2 and 3.

**Theorem 1.** *Let* $(\vec{X}, \mathcal{P}') = \mathsf{grm}(S, \emptyset)$ *and* $(\vec{Y}, \mathcal{P}) = \mathsf{grm}(T, \mathcal{P}')$. *Then,* $\mathsf{grm}$ *is a full abstract encoding, i.e.,* $S \sim_{\mathcal{T}} T$ *if and only if* $\vec{X} \sim_{\mathcal{P}} \vec{Y}$.

*Proof.* For the direct implication, assume that $S \sim_{\mathcal{T}} T$ and let $\mathcal{B}$ be a bisimulation for $S$ and $T$. Then, consider $\mathcal{B}' = \{(\mathsf{word}(S_0), \mathsf{word}(T_0)) \mid (S_0, T_0) \in \mathcal{B}\}$. Obviously, $(\mathsf{word}(S), \mathsf{word}(T)) \in \mathcal{B}'$. To prove that $\mathcal{B}'$ is a bisimulation, one assumes that $\mathsf{word}(S_0) \xrightarrow{a}_{\mathcal{P}} \vec{X}$ and proves that there exists $\vec{Y}$ such that $\mathsf{word}(T_0) \xrightarrow{a}_{\mathcal{P}} \vec{Y}$ with $(\vec{X}, \vec{Y}) \in \mathcal{B}'$. This proof is done by coinduction on the definition of $\mathsf{word}$, uses Lemmas 2, 3, and the definition of $\mathcal{B}'$.
For the reverse implication, assume that $\vec{X} \sim_{\mathcal{P}} \vec{Y}$, with $\vec{X} = \mathsf{word}(S)$ and $\vec{Y} = \mathsf{word}(T)$ and let $\mathcal{B}'$ be a bisimulation for $\vec{X}$ and $\vec{Y}$. Then, consider $\mathcal{B} = \{(S_0, T_0) \mid (\mathsf{word}(S_0), \mathsf{word}(T_0)) \in \mathcal{B}'\}$. Notice that $(S, T) \in \mathcal{B}$. The proof that $\mathcal{B}$ is a bisimulation, consists in showing that: given $(S_0, T_0) \in \mathcal{B}$, such that $S_0 \xrightarrow{a}_{\mathcal{T}} S_0'$, there exists $T_0'$ such that $T_0 \xrightarrow{a}_{\mathcal{T}} T_0'$ and $(S_0', T_0') \in \mathcal{B}$. The proof follows by rule coinduction on the LTS and uses Lemmas 2 and 3. □

Now we sketch the proof that pruning grammars also preserves bisimulation. We distinguish the grammars in the context through the subscript of $\sim$.

**Theorem 2.** $\vec{X} \sim_{\mathcal{P}} \vec{Y}$ *if and only if* $\vec{X} \sim_{\mathsf{prune}(\mathcal{P})} \vec{Y}$.

*Proof.* For the direct implication, the bisimulation for $\vec{X}$ and $\vec{Y}$ over $\mathcal{P}$ is also a bisimulation for $\vec{X}$ and $\vec{Y}$ over $\mathsf{prune}(\mathcal{P})$. For the reverse implication, if $\mathcal{B}'$ is a bisimulation for $\vec{X}$ and $\vec{Y}$ over $\mathsf{prune}(\mathcal{P})$, then $\mathcal{B} = \mathcal{B}' \cup \{(\vec{V}W, \vec{V}W\vec{Z}) \mid (W \to \vec{V}W\vec{Z}) \in \mathcal{P},\ W \text{ unnormed}\}$ is a bisimulation for $\vec{X}$ and $\vec{Y}$ over $\mathcal{P}$. □

*Correctness of the algorithm.* We now focus on the correctness of the function $\mathsf{bisimG}$. Before proceeding to soundness, we recall the *safeness property* introduced by Jančar and Moller [18].

**Lemma 4 (Safeness Property).** *Given a set of productions $\mathcal{P}$, $\vec{X} \sim_{\mathcal{P}} \vec{Y}$ if and only if the expansion tree rooted at $\{(\vec{X}, \vec{Y})\}$ has a successful branch.*

Notice that function $\mathsf{bisimG}$ builds an expansion tree by alternating between simplification—reflexive, congruence, and BPA—and expansion operations, as proposed by Jančar and Moller. These simplification rules are *safe* [18], in the sense that the application of any rule preserves the bisimulation from a parent node to at least one child node and, reciprocally, that bisimulation on a child node implies the bisimulation of its parent node.

While the safeness property is instrumental in proving soundness, the *finite witness property* is of utmost importance to prove completeness. This result follows immediately from the analysis by Jančar and Moller [18], which capitalizes on results by Caucal [5], and Christensen, Hüttel, and Stirling [6]:

**Lemma 5 (Finite Witness Property).** *Given a set of productions $\mathcal{P}$, if $\vec{X} \sim_{\mathcal{P}} \vec{Y}$ then the expansion tree rooted at $\{(\vec{X}, \vec{Y})\}$ has a finite successful branch.*

We refer to Caucal, Christensen, Hüttel, and Stirling for details on the proof of existence of a finite witness, as stated in Lemma 5. This proof is particularly interesting in that it highlights the importance of the BPA rules and of pruning productions on reaching such (finite) witness. The results in these two papers also elucidate the reason for the distinction, in the simplification phase, between the cases where all the symbols in the grammar are and are not normed (cf. program variable $\mathsf{rules}$ in function $\mathsf{expand}$). The safeness and finite witness properties ensure the termination of the algorithm, its soundness and completeness.

**Lemma 6 (Termination).** *Let $(\vec{X}, \mathcal{P}') = \mathsf{grm}(S, \emptyset)$ and $(\vec{Y}, \mathcal{P}) = \mathsf{grm}(T, \mathcal{P}')$. Then, the computation of $\mathsf{bisimG}(\vec{X}, \vec{Y}, \mathsf{prune}(\mathcal{P}))$ always terminates.*

*Proof.* Start by noticing that $\mathsf{prune}(\mathcal{P})$ always terminates. For $\mathsf{bisimG}$ itself, if $S \sim_{\mathcal{T}} T$ then, by Theorems 1 and 2, we have $\mathsf{word}(S) \sim_{\mathsf{prune}(\mathcal{P})} \mathsf{word}(T)$ and thus the existence of a finite successful branch is ensured by the finite witness property (Lemma 5). Hence, breadth-first search eventually terminates.

When $S \not\sim_{\mathcal{T}} T$, we easily conclude that all branches in the expansion tree are finite and thus $\mathsf{bisimG}(\vec{X}, \vec{Y})$ terminates. To conclude that all branches are finite, observe that any infinite branch is successful by definition and thus the safeness property would imply $\mathsf{word}(S) \sim_{\mathsf{prune}(\mathcal{P})} \mathsf{word}(T)$ and we would have $S \sim_{\mathcal{T}} T$, by Theorems 1 and 2. □

**Lemma 7.** *Let $(\vec{X}, \mathcal{P}') = \mathsf{grm}(S, \emptyset)$ and $(\vec{Y}, \mathcal{P}) = \mathsf{grm}(T, \mathcal{P}')$. If $\mathsf{bisimG}(\vec{X}, \vec{Y}, \mathsf{prune}(\mathcal{P}))$ returns **True**, then $\vec{X} \sim_{\mathsf{prune}(\mathcal{P})} \vec{Y}$.*

*Proof.* Function $\mathsf{bisimG}$ returns **True** whenever it reaches a (finite) successful branch in the expansion tree rooted at $\{(\vec{X}, \vec{Y})\}$, i.e., a branch terminating in an empty node. Conclude with the safeness property, Lemma 4. □

From the previous results, the soundness of our algorithm is now immediate: the algorithm to check the bisimulation of context-free session types is sound with respect to the meta-theory of context-free session types.

**Theorem 3 (Soundness).** *Let* $(\vec{X}, \mathcal{P}') = \mathsf{grm}(S, \emptyset)$ *and* $(\vec{Y}, \mathcal{P}) = \mathsf{grm}(T, \mathcal{P}')$. *If* $\mathsf{bisimG}(\vec{X}, \vec{Y}, \mathsf{prune}(\mathcal{P}))$ *returns* **True** *then* $S \sim_{\mathcal{T}} T$.

*Proof.* From Theorem 1, Theorem 2, and Lemma 7. □

Given that the algorithm terminates (Lemma 6), we know that if $S \not\sim_{\mathcal{T}} T$, then $\mathsf{bisimG}(\vec{X}, \vec{Y}, \mathsf{prune}(\mathcal{P}))$ returns **False**, where $(\vec{X}, \mathcal{P}') = \mathsf{grm}(S, \emptyset)$ and $(\vec{Y}, \mathcal{P}) = \mathsf{grm}(T, \mathcal{P}')$. We now show that the algorithm to check the bisimulation of context-free session types is complete with respect to the meta-theory of context-free session types. The finite witness property is paramount to achieve this result.

**Theorem 4 (Completeness).** *Let* $(\vec{X}, \mathcal{P}') = \mathsf{grm}(S, \emptyset)$ *and* $(\vec{Y}, \mathcal{P}) = \mathsf{grm}(T, \mathcal{P}')$. *If* $S \sim_{\mathcal{T}} T$ *then* $\mathsf{bisimG}(\vec{X}, \vec{Y}, \mathsf{prune}(\mathcal{P}))$ *returns* **True**.

*Proof.* Assume $S \sim_{\mathcal{T}} T$. By Theorems 1 and 2, we have $\vec{X} \sim_{\mathsf{prune}(\mathcal{P})} \vec{Y}$. Hence, Lemma 5 ensures the existence of a finite successful branch on the expansion tree rooted at $\{(\vec{X}, \vec{Y})\}$, i.e., a branch terminating in an empty node. Since our algorithm traverses the expansion tree using breadth-first search it will, eventually, reach the empty node and conclude the bisimulation positively. □

Theorem 4 ensures that if $\mathsf{bisimG}(\vec{X}, \vec{Y}, \mathcal{P})$ returns **False** then $S \not\sim_{\mathcal{T}} T$.

## 5 Evaluation

This section discusses the behaviour of our algorithm in the real world. Both for testing and for performance evaluation, we require test suites. We started with a carefully crafted, manually produced, suite of valid and invalid tests. This test suite was assembled by gathering pairs of types that emerged from examples we have studied and from programs we have written in FreeST, a programming language with context-free session types [2]. The tests produced by this method are, on the one hand, small, and, on the other hand, lacking diversity.

We then turned our attention to the automatic generation of test cases. Producing pairs of arbitrary (well-formed) types that share no variables is simple. However, the probability that a randomly generated pair of types turns out to be bisimilar is extremely low. For this reason, we generate arbitrary pairs of types that are bisimilar by construction. Theorem 5 naturally induces an algorithm: given a natural number $n$ (the size of the pair), arbitrarily select for the base case ($n = 0$) one of the pairs in item 1 of the theorem and for the recursive case ($n \geq 1$) one of the pairs in 2–12 items.

**Theorem 5 (Properties of type bisimilarity).**

1. $\mathsf{skip} \sim_{\mathcal{T}} \mathsf{skip}$ *and* $\sharp B \sim_{\mathcal{T}} \sharp B$;
2. $S;T \sim_{\mathcal{T}} U;V$ *if* $S \sim_{\mathcal{T}} U$ *and* $T \sim_{\mathcal{T}} V$;
3. $\mu X.S \sim_{\mathcal{T}} \mu X.T$ *if* $S \sim_{\mathcal{T}} T$;
4. $\star\{\ell_i\colon S_i\}_{i\in I} \sim_{\mathcal{T}} \star\{\ell_i\colon T_i\}_{i\in I}$ *if* $(S_i \sim_{\mathcal{T}} T_i)_{i\in I}$;
5. $S \sim_{\mathcal{T}} T;\mathsf{skip}$ *and* $S \sim_{\mathcal{T}} \mathsf{skip};T$ *if* $S \sim_{\mathcal{T}} T$;
6. $\star\{\ell_i\colon S_i\}_{i\in I};U \sim_{\mathcal{T}} \star\{\ell_i\colon T_i;V\}_{i\in I}$ *if* $(S_i \sim_{\mathcal{T}} T_i)_{i\in I}$ *and* $U \sim_{\mathcal{T}} V$;
7. $T \sim_{\mathcal{T}} S$ *if* $S \sim_{\mathcal{T}} T$;
8. $R;(S;T) \sim_{\mathcal{T}} (U;V);W$ *if* $R \sim_{\mathcal{T}} U$, $S \sim_{\mathcal{T}} V$, *and* $T \sim_{\mathcal{T}} W$;
9. $\mu X.\mu Y.S \sim_{\mathcal{T}} \mu X.[X/Y]T \sim_{\mathcal{T}} \mu Y.[Y/X]T$ *if* $S \sim_{\mathcal{T}} T$;
10. $\mu X.S \sim_{\mathcal{T}} T$ *if* $S \sim_{\mathcal{T}} T$ *and* $X \notin \mathsf{free}(S)$;
11. $[U/X]S \sim_{\mathcal{T}} [V/X]T$ *if* $S \sim_{\mathcal{T}} T$ *and* $U \sim_{\mathcal{T}} V$;
12. $\mu X.S \sim_{\mathcal{T}} [\mu X.T/X]T$ *if* $S \sim_{\mathcal{T}} T$.

*Proof.* 1–3: Bisimulation is a congruence. 4–12: Thiemann and Vasconcelos [26] exhibit the appropriate bisimulations. □

For evaluating the algorithm on non-bisimilar pairs we add the following five anti-axioms to the list in Theorem 5: (1) $\mathsf{skip} \not\sim_{\mathcal{T}} \sharp B$; (2) $?B \not\sim_{\mathcal{T}} !B$; (3) $\mathsf{skip} \not\sim_{\mathcal{T}} \star\{\ell_i\colon S_i\}_{i\in I}$; (4) $\oplus\{\ell_i\colon S_i\}_{i\in I} \not\sim_{\mathcal{T}} \&\{\ell_i\colon S_i\}_{i\in I}$; (5) $\star\{\ell_i\colon S_i\}_{i\in I} \not\sim_{\mathcal{T}} \star\{\ell_j\colon S_j\}_J$ where $I \subset J$. We generate two types using the same methodology as for the positive case and, then, discard the data collected when the pair turns out to be bisimilar. This produces pairs of types that are much closer than those obtained by random generation, thus hopefully approaching the reality that the compilers face when in production.

We used QuickCheck [7] to generate two test suites. That for bisimilar pairs is constructed based on Theorem 5, whereas the construction of non-bisimilar tests relies on Theorem 5 plus the anti-axioms above. Both test suites comprise 2000 entries, featuring types with a number of nodes (in the syntax tree) ranging from 1 to 200.

The base algorithm described in the previous section turns out to behave quite poorly. We then implemented the following variants.

1. *Eliminating redundant productions in the grammar.* Since the size of the expansion tree depends, among other things, on the number of productions in the grammar, generating smaller grammars seems a promising optimisation. Rather than blindly adding a new production $Y \to \vec{Z}$ to the grammar (in function $\mathsf{word}$, Section 3), we look, in the set of productions, for a production $W \to \vec{X}$ syntactically equal to the former, up to renaming of non-terminal symbols. In this case, we add no new production and return non-terminal $W$ instead. To find $W$, we look for the least fixed-point of the transitions in the languages generated by $\vec{Z}$ and $\vec{X}$ and compare them. This optimisation does not compromise the results of soundness, completeness, nor termination.
2. *Using a filter rule that removes nodes with hopeless pairs.* A filter rule ensures that nodes composed by pairs of types with different norms (if normed) are removed from the expansion tree, since these types are not bisimilar. The filter rule preserves the results of soundness, completeness, and termination.

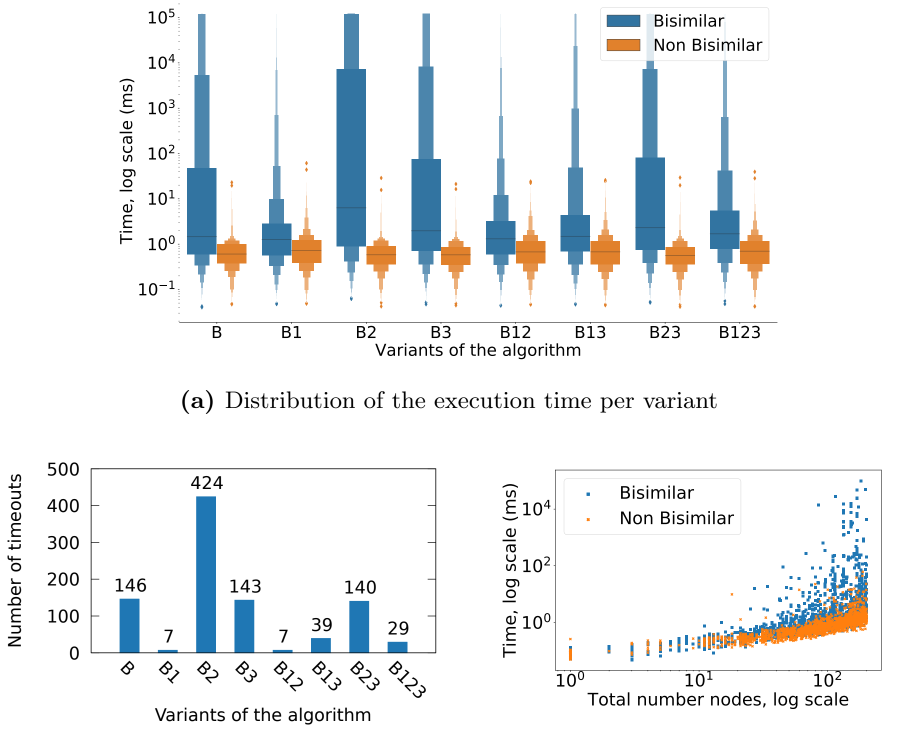

**(a)** Distribution of the execution time per variant

**(b)** Number of timeouts per variant

**(c)** Runtime of B1 per number of nodes

**Fig. 2:** Results on the test suite composed by bisimilar pairs of types is represented in blue and the test suite with non-bisimilar pairs is represented in orange. Time is in milliseconds. Scales of 2a and 2c are logarithmic; scale of 2b is linear.

3. *Using a double-ended queue to prepend promising children.* A double-ended queue allows prioritizing nodes with potential to reach an empty node faster. The algorithm prepends (rather than appends) empty nodes or nodes whose pairs $(\vec{X}, \vec{Y})$ are such that $|\vec{X}| \leq 1$ and $|\vec{Y}| \leq 1$. This procedure does not compromise soundness, completeness, nor termination because the number of terminal symbols is finite and the algorithm takes advantage of the reflexive and congruence rules to remove previously visited nodes from the queue.

To better understand how the algorithm performs in practice, we tested all the optimisations and their combinations. We evaluate each variant 1–3 individually (denoted by B1–B3) and all their combinations. For instance, B12 denotes the variant obtained from combining optimisations 1 and 2 above. B stands for the base algorithm, $\mathsf{bisimT}$. We implemented the base algorithm and its variants in Haskell, using the Glasgow Haskell Compiler (version 8.6.5). The evaluation was conducted on a machine with an Intel Core i7-6700K at 4.2GHz and 8 GB of RAM running Arch Linux; tests were run under a timeout of 2 minutes.

Figure 2a depicts the distribution of the execution times (in $ms$) for both test suites and all variants. We observe that the behavior of negatives tests is roughly the same in all variants. However, the execution time for the positive tests differ from variant to variant. These differences mainly depend on the trade-off between the computational effort required for each optimisation and the efficiency they bring to deciding the equivalence of grammars. We observe that including optimisation 1 improves the execution time, while the rest, in general, does not. The combination of optimizations has a positive impact on execution time, with the exception of the B23 variant, whose distribution is worse than the base case.

Figure 2b shows the number of timeouts for each variant. The base case, B, has 146 positive tests whose execution time exceeds 2 minutes. The distribution of timeouts per variant exhibits a behavior that is consistent with that of runtime shown in Figure 2a. All combinations lead to a reduction in the number of timeouts, when compared to the base case.

Variant B1, resulting from considering optimisation 1, performs better than all others, presenting a median of 1.4 milliseconds and 7 timeouts, both for the positive tests. By taking advantage of optimisation 1, the number of timeouts reduced by 95%. The remaining positive tests take, on average, 1863.38 ms to complete with the base algorithm and 195.68 ms with variant B1, resulting in an 89% reduction in the execution time. This is the variant in production for the FreeST compiler [2].

The distribution of the execution time of B1 against the size of the input types is depicted in Figure 2c. As expected, the execution time increases considerably with the number of nodes. Although we have carried out tests with a fairly large number of nodes in the abstract syntax trees, we remark that, when used in a compiler, the algorithm will mostly come across types with a reduced number of nodes.

## 6 Conclusion

Context-free session types are a promising tool to describe protocols in concurrent programs. In order to be incorporated in programming languages and effectively used in compilers, a practical algorithm to decide bisimulation is called for. Taking advantage of a process algebra graph representation of types to decide bisimulation [12,13], we developed one such algorithm and proved it correct. The algorithm is incorporated in a compiler for a concurrent functional language equipped with context-free session types [2].

Possible extensions to this work include addressing higher-order session types. We also plan to extend the implementation of the algorithm to cope with context-free grammars in Greibach Normal Form that are not necessarily deterministic.

*Acknowledgements.* We thank Alcides Fonseca for helping with the testing process, and Filipe Casal, Alexandra Silva, and Peter Thiemman for comments and discussions. This work was supported by FCT through the LASIGE Research Unit, ref. UIDB/00408/2020 and by Cost Action CA15123 EUTypes.
